# Vacuum-Assisted Continuous Circular Capsulorhexis Using Bimanual Irrigation and Aspiration System of Phaco Machine in Immature Cataract

**DOI:** 10.1155/2013/921646

**Published:** 2013-11-04

**Authors:** Huseyin Oksuz, Mutlu C. Daglioglu, Mesut Coskun, Özgür İlhan, Esra Ayhan Tuzcu, Nilufer Ilhan, Emre Ayıntap, Uğurcan Keskin, İbrahim Taşkın

**Affiliations:** ^1^Department of Ophthalmology, Medical Faculty, Mustafa Kemal University, 31400 Hatay, Turkey; ^2^Department of Ophthalmology, Medical Faculty, Bezmialem Vakif University, Istanbul, Turkey; ^3^Private Maya Eye Center, Adana, Turkey

## Abstract

Seventy-eight eye of 65 patients were enrolled in this retrospective clinical study. Two-side ports are made with a 23-gauge stiletto knife. The irrigation handpiece is introduced into the anterior chamber through one side port and 27-gauge cystotome is introduced through the other one. Anterior capsular flap is created with cystotome. The capsular flap is vacuumed with a 25-gauge visco elastic's cannula, which connected to the phaco machine vacuum. The continuous circular capsulorhexis (CCC) is completed with the using bimanual irrigation and aspiration system of phaco machine. Vacuum-assisted CCC technique was used in 78 cases of uncomplicated immature senile cataracts. All cases were done under sub-Tenon's anesthesia. A complete CCC was achieved in all cases. Performing CCC with our technique is easy, safe, and cheap. It may be an alternative method to CCC by using OVD and forceps.

## 1. Introduction 

The continuous circular capsulorhexis (CCC) has rapidly increased in popularity as the procedure of choice when using phacoemulsification for cataract extraction [1]. CCC can be performed in several ways. It can be performed using cystotome, small incision capsulorhexis forceps, or utrata forceps [1–4]. When we perform CCC with these tools we use ophthalmic viscosurgical devices (OVDs). We describe a new technique for CCC using bimanual irrigation and aspiration system of the phaco machine without using OVD and forceps. The aim of this study was to evaluate the efficacy and safety of CCC performing technique.

## 2. Methods 

**Figure 1:**
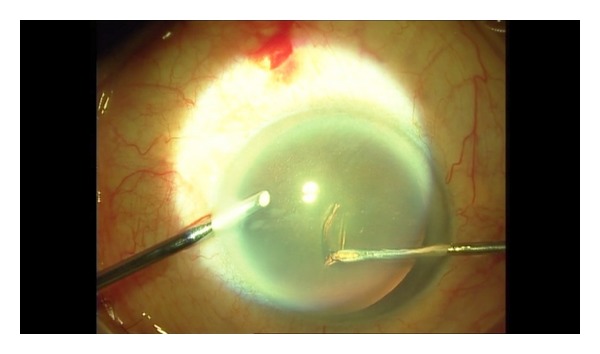
Anterior capsular flap is created with cystotome.

**Figure 2:**
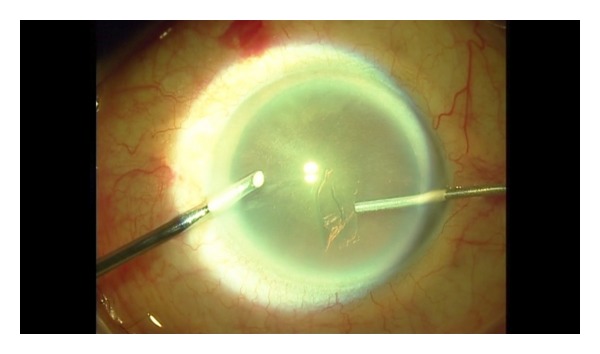
The capsular flap is vacuumed with 25-gauge visco elastic's cannula.

**Figure 3:**
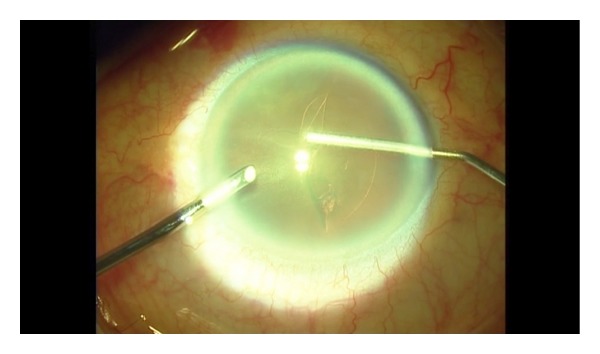
The capsular flap is vacuumed with 25 gauge a visco elastic's cannula.

**Figure 4:**
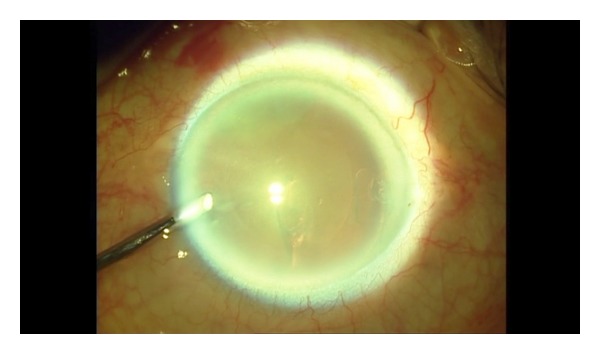
After CCC completed.

**Figure 5:**
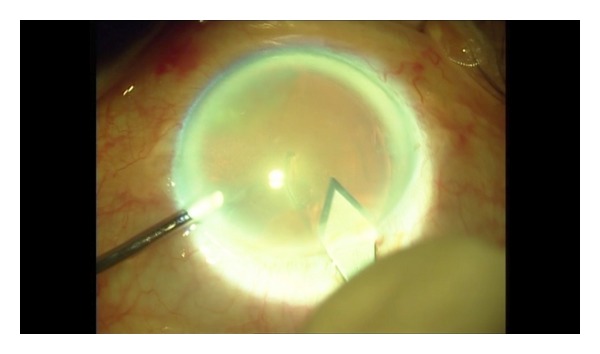
Clear corneal tunnel incision is made with 2.75 metal blade under continuous irrigation.

The study was carried out at Mustafa Kemal University from May 2011 to January 2012. Preoperative ocular examinations included Snellen visual acuity, biomicroscopic examination, Goldmann applanation tonometry, axial length, and anterior chamber depth measurements with A-scan ultrasonography. Written informed consent was obtained from each patient. Institutional review board approval was obtained through the University of Gaziantep Faculty of Medicine. Patient with any other ocular disease, such as lens-induced uveitis, or glaucoma, or with a history of ocular trauma were excluded. Cyclopentolate 1% and phenylephrine 2,5% eye drops were used for mydriasis four times 1 hour before the surgery. A single surgeon (H.O.) performed all the surgical procedures. After sub-Tenon's anesthesia with lidocaine 1 cc 1%, the eyes were prepared and draped. Two side ports are made with 23 gauge stiletto knife at 9 o'clock and 2 o'clock. The irrigation hand piece is introduced into the anterior chamber through 2 o'clock side port with irrigation on. The irrigation hand piece is attached balanced salt solution with bottle height 90 cm above eye level. 27-gauge cystotome is introduced through 9 o'clock side port. Anterior capsular flap is created with cystotome. The capsular flap is vacuumed with a 25-gauge visco elastic's cannula, which is connected to the phaco machine vacuum (Figures 1, 2, 3, 4, and 5). The phaco machine vacuum setting at this stage is 400 mm Hg. CCC of approximately 5 mm was made. During this period, the irrigation hand piece held in the left hand maintains deep anterior chamber and stabilizes the globe. Clear corneal tunnel incision is made with 2.75 metal blade under continuous irrigation. Hydrodissection was performed through main incision. After hydrodissection 0.1 cc OVD was injected into the anterior chamber for protection corneal endothelial cells during phacoemulsification. Phacoemulsification was performed in the capsular bag, and cortical lens material was aspirated with a bimanual irrigation/aspiration cannula, the capsular bag was expanded with the OVD, and a foldable IOL was implanted into the capsular bag. The OVD was aspirated from the retrolental space, the capsule fornix, and the anterior chamber using a bimanual irrigation/aspiration cannula. The corneal incision was checked for water tightness and left unsutured. Postoperatively, all patients used ofloxacin 0.3% and prednisolone acetate 1% eye drops four times a day for 1 month. On the first postoperative day, corneal appearance, intraocular pressure, and postoperative complications were analyzed. Postoperative examinations were done at 1 day, 1 weeks, 1 and 3 months.

## 3. Results

We have used this technique in 78 eyes uncomplicated immature senile cataracts. All cases were done under sub-Tenon's anesthesia. The mean age was 65,42 ± 12,20 years. Preoperative mean intraocular pressure (IOP) is 14,42 ± 4,32 mm Hg and 20,54 ± 5,16 mm Hg on first postoperative day. The difference was significant (*P* <0,05, according to the Mann-Whitney *U* test). The IOP then gradually decreased to preoperative levels by one week postoperativly. A complete capsulorhexis was achieved in all cases. All surgeries went uneventful. At 1 day postoperative visit, corneal edema was present in 2 (2.3%) eyes. It resolved 1 week after surgery.

## 4. Discussion

Cataract surgery is one of the most performed surgeries. Cataract operations are generally performed by phacoemulsification technique. The CCC is one of the prerequisites for successful phacoemulsification. When performing CCC surgeons use either viscoelastics and forceps or a needle connected to the infusion bottle raised high enough to give adequate depth [2–4]. In our technique, we prefer to use irrigation hand piece to achieve well maintained deep anterior chamber and relaxed zonules throughout the capsulorhexis. Especially while performing CCC, the surgeon must be ready to immobilize the globe with second instrument. The globe should be stabilized by holding from conjunctiva with a forceps. Holding conjunctiva with forceps can lead to hemorrhage, tear, and increased patient discomfort. This is undesirable, especially under topical anesthesia. These problems can be overcome by using the above-described technique. This method is appropriate for cataract surgery under topical anesthesia.

Sethi et al. performed CCC with the anterior chamber maintainer using cystotome [4]. Performing CCC with such a method causes capsular flap waving due to irrigation. Capsular flap should be hold and released 2 to 3 times performing CCC. Capsule should be bent at each time. It is difficult to release and catch the capsule. This prolongs the procedure time. There is no necessity to bend the capsule in our technique. Additionally, it is easy to hold and release the capsule with an aspiration cannula. While performing CCC under anterior chamber maintainer, some air emboli may occur into anterior chamber. In this case air should be aspirated from there and CCC be completed afterwards. In our technique, even in a situation of air emboli, air could be aspirated with an aspiration cannula without any delay. Anterior chamber should have adequate depth to perform CCC in our method. To do so, bottle height should be 90 to 120 cm at least. There should be no liquid leak from side port incision.

Sallet did CCC under anterior chamber maintainer by using microforceps [5]. Microforceps may not be available at any clinic. Our technique requires only a 25-gauge viscoelastic cannula. Sallet did not use viscoelastic material while performing CCC and IOL implantation under anterior chamber maintainer. There was no significant difference in corneal endothelial cell count between patients operated with or without viscoelastic material.

We used 0.1 mL viscoelastic material after hydrodissection. We applied small amount of OVD for protection of the endothelium during phacoemulsification. The reason to use it was not to harm corneal endothelial cells during phacoemulsification. Yet we have not experienced any corneal edema due to endothelial failure. 0.5 mL OVD is being applied only to one eye during CCC by using viscoelastic material. This amount is five times more than we used. Should CCC be performed by using our technique in clinics with high concentrations of cataract surgery, OVD consumption and operation costs may diminish.

Viscoelastic material may leak while performing CCC by using OVD and utrata forceps. A reinjection of OVD may necessitate. Shallowing may occur at anterior chamber due to OVD leak and tear in capsule may move more peripherally, and leaving CCC uncompleted. Anterior chamber stays more stable and CCC is completed without complications at every case with our technique.

CCC should be done quickly with this technique. If procedure exceeds then slight corneal edema may occur. When corneal edema is healed with 2,75 mm main phaco incision. The time of procedure lessens as operator gains experience. Temporary corneal edema may occur due to increased hydrostatic pressure. Whenever the pressure vanishes then corneal edema alleviates. Liquid in the bottle should not finish. Amount of the liquid should always be controlled.

There is growing trend toward microincision cataract surgery techniques [6]. Creating CCC through a small incision using traditional capsulorhexis forceps can be difficult. Traditional based-hinged capsulorhexis forceps fit too tightly inside the main incision, reducing maneuverability and putting local stress on the wound, which can distort the corneal surface and reduce visualization.

Microforceps should be used while doing CCC in microincision cataract surgery. Our technique enables to perform CCC without using microforceps.

This method may suit in patients with white cataract and undergoing CCC. In white cataract, the anterior capsule could be perforated with cystotome and a milky material fills in anterior chamber. This upset the anterior chamber sight. CCC is performed after cleaning the milky material. During procedure anterior chamber may get shallow and cause tears in capsule. However, in our technique, even milky material fills the anterior chamber, it is aspirated immediately and the anterior chamber is not dimmed. Moreover, the anterior chamber is left more stabilized. This technique may be tried in white cataract while performing CCC.

An additional advantage of our technique may be that the capsule is not visualized thoroughly under viscoelastic material but much more visible under liquid. This may be attributed to the refractive index of viscoelastic material. Performing CCC with our technique is not suitable for patients with subluxed lens. In mininuc cataract surgery the CCC is performed through side port incision using cystotome and an anterior chamber maintainer [2, 3].

## 5. Conclusion

We think performing CCC with our technique is easy, safe, and cheap. It may be an alternative method to CCC by using OVD and forceps. However, more studies with large number of patients have to be carried out.
